# Conscious awareness of a visuo-proprioceptive mismatch: Effect on cross-sensory recalibration

**DOI:** 10.3389/fnins.2022.958513

**Published:** 2022-08-31

**Authors:** Anna Hsiao, Trevor Lee-Miller, Hannah J. Block

**Affiliations:** Department of Kinesiology, School of Public Health, Indiana University Bloomington, Bloomington, IN, United States

**Keywords:** proprioception, recalibration, multisensory, peripersonal space, visuo-proprioceptive conflict, causal inference, cross-sensory calibration, hand

## Abstract

The brain estimates hand position using vision and position sense (proprioception). The relationship between visual and proprioceptive estimates is somewhat flexible: visual information about the index finger can be spatially displaced from proprioceptive information, resulting in cross-sensory recalibration of the visual and proprioceptive unimodal position estimates. According to the causal inference framework, recalibration occurs when the unimodal estimates are attributed to a common cause and integrated. If separate causes are perceived, then recalibration should be reduced. Here we assessed visuo-proprioceptive recalibration in response to a gradual visuo-proprioceptive mismatch at the left index fingertip. Experiment 1 asked how frequently a 70 mm mismatch is consciously perceived compared to when no mismatch is present, and whether awareness is linked to reduced visuo-proprioceptive recalibration, consistent with causal inference predictions. However, conscious offset awareness occurred rarely. Experiment 2 tested a larger displacement, 140 mm, and asked participants about their perception more frequently, including at 70 mm. Experiment 3 confirmed that participants were unbiased at estimating distances in the 2D virtual reality display. Results suggest that conscious awareness of the mismatch was indeed linked to reduced cross-sensory recalibration as predicted by the causal inference framework, but this was clear only at higher mismatch magnitudes (70–140 mm). At smaller offsets (up to 70 mm), conscious perception of an offset may not override unconscious belief in a common cause, perhaps because the perceived offset magnitude is in range of participants’ natural sensory biases. These findings highlight the interaction of conscious awareness with multisensory processes in hand perception.

## Introduction

Where we perceive our hands in space has a substantial impact on how we carry out manual tasks. For example, when hammering a nail steadied by the thumb and index finger, misjudging the nail’s position could result in injured fingers. Through proprioception, the brain can estimate hand or finger position using signals from the muscles, joints, and skin even in the absence of vision (Proske and Gandevia, 2012). Visual and proprioceptive position estimates have different variances and biases due to independent processing in the visual and proprioceptive systems, thus they are unlikely to agree perfectly (Smeets et al., 2006). The brain is thought to weight and combine available unimodal estimates, resulting in a single multisensory estimate that minimizes variance (Ghahramani et al., 1997; Ernst and Banks, 2002; Beauchamp et al., 2010). This has been observed across several human behaviors and sensory modality combinations (van Beers et al., 1999; Ernst and Banks, 2002; Sober and Sabes, 2005; Kording and Wolpert, 2006; Fetsch et al., 2013; Blouin et al., 2014).

When there is an externally-imposed spatial offset between available sensory cues, cross-sensory recalibration has been observed, where one or both unimodal estimates shifts toward the other (Noppeney, 2021). For example, a person viewing a representation of their hand that is offset from true hand position is likely to shift their proprioceptive estimate of hand position toward the visual estimate. This has been observed in studies of rubber hand illusion (RHI) (Botvinick and Cohen, 1998; Samad et al., 2015; Fang et al., 2019) and visuomotor adaptation (Henriques and Cressman, 2012; Salomonczyk et al., 2013; Rossi et al., 2021), both of which involve a spatial visuo-proprioceptive mismatch. When both visual and proprioceptive estimates are assessed, evidence suggests that both estimates shift toward each other in the presence of a mismatch (van Beers et al., 2002; Block and Bastian, 2011; Munoz-Rubke et al., 2017; Mirdamadi et al., 2021).

While there is ample experimental evidence for cross-sensory recalibration, the principles by which it operates are unclear, and may depend on the task and context (Noppeney, 2021). The framework of causal inference is likely relevant to many aspects of multisensory processing, including cross-sensory recalibration (Wei and Körding, 2011). According to this framework, sensory cues that are perceived to have a common cause are more likely to be integrated, compared to cues that are perceived to belong to separate causes (Kording et al., 2007; Shams and Beierholm, 2010; French and DeAngelis, 2020). Studies with various paradigms have supported the idea that the smaller the spatial disparity between two stimuli, the more often people perceive them as having a common cause (Kording et al., 2007; French and DeAngelis, 2020).

Some studies have suggested that perceiving a mismatch or conflict between cues affects cross-sensory recalibration in visual-auditory localization (Kording et al., 2007) as well as visuo-proprioceptive localization (Samad et al., 2015). Knowledge or awareness of a mismatch influences causal inference by serving as a Bayesian prior (Debats and Heuer, 2018). Prior belief that two cues belong to separate causes, which may be influenced by directing attention toward a mismatch, reduces the likelihood of integration (Noppeney, 2021). Within the model, the degree to which awareness of a separate cause affects cross-sensory recalibration is not fully known (Berger and Bülthoff, 2009).

One open question concerns the role of the magnitude of the cue mismatch. Prism exposure studies, in which the visual field is offset from the proprioceptive cue of hand position, have suggested that cross-sensory recalibration is affected by knowledge of the offset only if the offset is relatively large (Welch and Warren, 1980). This is not consistent with the causal inference framework (French and DeAngelis, 2020). Even when the cue mismatch is large (20° of prismatic shift) and visuo-proprioceptive unity is no longer perceived, substantial recalibration of both visual and proprioceptive estimates still occur (Welch and Warren, 1980). However, prism studies are somewhat limited in that prisms shift the whole visual field, not just the visual cue of the hand, and can cause visual distortions.

In the present study we build on this literature by assessing visuo-proprioceptive recalibration in response to a gradually imposed visuo-proprioceptive mismatch. Experiment 1 asked how frequently a gradually-imposed 70 mm visuo-proprioceptive mismatch (Munoz-Rubke et al., 2017; Mirdamadi et al., 2021) is consciously perceived, and whether such awareness was linked to reduced visuo-proprioception recalibration, as predicted by the causal inference model (French and DeAngelis, 2020). Experiment 2 tested a larger displacement of 140 mm and asked participants about their perception more frequently. We predicted that the participants who reported the greatest proportion of the true offset would recalibrate the least, in line with the causal inference model. Finally, Experiment 3 tested whether participants were biased at estimating the lengths of lines projected in the 2D virtual reality display, to control for the possibility that participants under-report the magnitude of the true offset because they tend to underestimate distances in general.

## Materials and methods

### Participants

A total of 96 healthy adults participated in this study, which consisted of three experiments. Experiment 1 participants each completed two sessions on different days. Experiments 2 and 3 comprised a single session each. 62 (34 female, mean age 21.8 years, SD 4.2) participated in Experiment 1. Twenty (17 female, mean age 20.9 years, SD 4.2) participated in Experiment 2. Twenty (13 female, mean age 22 years, SD 4.3) participated in Experiment 3. Six participants participated in both experiments 2 and 3. All participants reported being right-handed. All reported normal or corrected-to-normal vision, and no known neurological or musculoskeletal conditions. The study was approved by the Indiana University Institutional Review Board. All participants gave written informed consent before participating in the study.

### Experiment 1

Participants completed two sessions each, on different days at least 5 days apart: a Mismatch session and a Veridical session. Session order was counterbalanced across participants. Time between sessions was 14.4 ± 13.8 days (mean ± SD).

#### Apparatus

**FIGURE 1:**
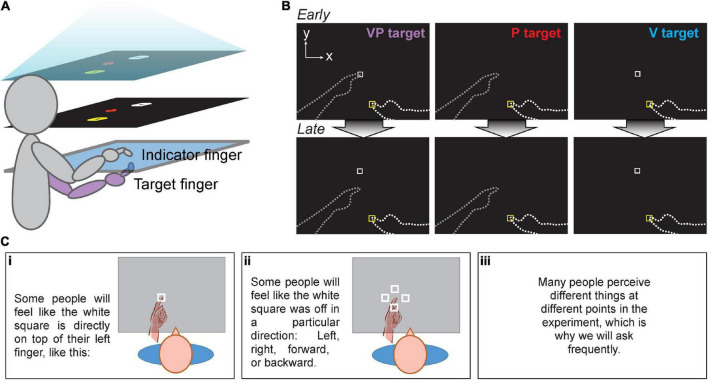
**(A)** Apparatus for all three experiments. Task display (top) was viewed in a mirror (middle), making it appear that images in the mirror were in the plane of the touchscreens (bottom). For Expt. 1 and 2, the right index finger served as the indicator finger, always remaining above the glass, and the left as the target finger, always remaining below the glass. Participants had no direct vision of either hand. **(B)** Targets in Expt. 1 and 2. Participants moved their indicator finger from the yellow start box to the perceived VP, P, or V target position. No performance feedback was given. Top row: Early in the session, VP targets were veridical, with the white square projected directly over the target fingertip. Bottom row: The white square gradually shifted forward from the target fingertip to create visuo-proprioceptive offset in Expt. 2 and the Mismatch session of Expt. 1. Dashed lines not visible to participants. **(C)** Experiment 2 participants received specific instructions in a slide presentation before beginning the task. This sequence was intended to prepare participants to report their perceived visuo-proprioceptive offset after each block of 21 trials, without revealing that there would be an externally imposed offset.

Participants sat at a 2D reflected rear projection apparatus composed of two touchscreen frames (PQ Labs) with a 3-mm-thick pane of glass in between. The touchscreens utilized infrared beams to detect touch with <0.5 mm resolution. Participants viewed the task display in a horizontal mirror positioned just below eye-level. This resulted in images that appeared to be in the plane of the touchscreens while also preventing the participants from seeing their hands (Figure 1A). The total display area was 75 cm × 100 cm. Black fabric draped around the participant’s shoulders obscured their view of their upper arms and the surrounding room. Participants kept their left hand (target) below the touchscreen during the experiment and on their lap when not needed, while their right (indicator) hand remained above the touchscreen and below the mirror.

#### Targets

Participants were asked to use their right index finger to indicate the perceived position of a series of three different target types related to the left (target) index finger: proprioceptive (P), visual (V), and visuo-proprioceptive (VP) targets. The V target was a projected image of a white box, and the P target was the participant’s target index finger placed on a tactile marker beneath the touchscreen glass (Figure 1B). There were two possible target positions, about 33 and 36 cm in front of the participant’s chest, 4 and 7 cm left of body midline. The VP target included both the target finger and the white box and was used to create the visuo-proprioceptive mismatch. The V and P targets were used to assess visual and proprioceptive recalibration, respectively: when the VP target has a forward offset of the visual component, overshoot of P targets represents proprioceptive recalibration and undershoot of visual targets represents visual recalibration.

#### Single trial procedure

Participants began each trial by placing their indicator finger in the starting position above the glass, as indicated by a 1 cm yellow box. To help the participant reach the start position, a 0.8 cm blue dot appeared when the indicator finger was in contact with the touchscreen glass and positioned near the yellow start box. The yellow start box could appear in any of five locations, arranged like a plus sign at the participant’s midline, about 15 cm in front of the chest. The blue dot disappeared as soon as the indicator finger left the start box, preventing participants from having online or endpoint feedback about indicator finger position.

Once the indicator finger was correctly positioned, participants heard an audio cue instructing them to keep their eyes on a red cross that appeared at a random position within 10 cm of the target. However, eye movements were not recorded or enforced, and this was not intended to override subjects’ instinctive saccades to target position. The red cross was included in this paradigm (Block and Bastian, 2011, 2012; Block et al., 2013; Munoz-Rubke et al., 2017; Liu et al., 2018; Block and Sexton, 2020; Mirdamadi et al., 2021) to discourage conscious strategies involving gaze, particularly on P targets. In other words, we wanted to avoid having some subjects fixate where they think the P target is, and others staring off into space on P targets.

Next, participants were instructed to place their target finger on one of the tactile markers (P or VP target), or to rest their target hand in their lap (V target), and the white box appeared in the display (V or VP target). Finally, participants heard a beep, cueing them to begin the trial. For VP trials, participants were told during task training that the white box would appear directly over their target fingertip and that they should place their indicator finger at that location.

Participants were trained to lift their indicator finger off the glass from the starting position and place it down where they thought the target was positioned, without dragging their finger along the glass. Participants were notified that there were no speed requirements, and that adjustment was allowed. Once the participants had their indicator index finger on their estimated target position for 2 s, this position was recorded as the final estimate and the trial concluded.

Certain aspects of the procedure were intended to prevent motor adaptation of the indicator hand, allowing us to assess changes in perception of the target hand. Multiple start and target positions were used and randomized to prevent memorization, and no performance feedback or knowledge of results was given. Thus, participants had no information about the accuracy of their indicator finger placements in relation to the target (for review see: Shadmehr et al., 2010). In addition, participants were instructed to reach at a comfortable pace, to adjust if needed, and not to rush.

#### Sessions

Each session began with a baseline of veridical targets, followed by a single block of 21 V, 21 P, and 42 VP trials in the order V, VP, P, VP. In the Mismatch session, visuo-proprioceptive offset was imposed gradually by shifting the white square 1.67 mm forward after each VP trial (every two trials), to a maximum offset of 70 mm at the end of the single block of 84 trials. In the Veridical session, no offset was imposed, and the white square remained over the target finger throughout. At the end of each session, participants were asked to rate their attention level, quality of sleep the prior night, and fatigue caused by the experiment on a scale of 1–10.

#### Instructions

At the end of each session, participants were asked *“Did it always feel like the white square was directly on top of your left finger, or did it feel off?”* If participants replied with “it felt off,” they were then asked in what direction the white square felt offset from the left finger, and by how much at most. Participants were permitted to estimate this magnitude in either centimeters or inches. This approach was chosen to be consistent with previous studies using the visuo-proprioceptive recalibration paradigm (Block and Bastian, 2011; Munoz-Rubke et al., 2017; Mirdamadi et al., 2021).

#### Data analysis

We used a χ^2^ test to compare across sessions the proportion of participants that perceived a forward offset (compared to no offset or any offset direction other than forward). In the Mismatch session, visual and proprioceptive recalibration (Δŷ_*V*_ and Δŷ_*P*_) were calculated as we have done in previous studies with this task structure (Block and Bastian, 2011, 2012; Block et al., 2013), subtracting indicator finger endpoint y-dimension positions on the first four V or P trials of the 84-trial block from the last four:


(1) $$\Delta \,{\hat{y}}_{V}=70-\left(l\,a\,s\,t\,4\,V\,e\,n\,d\,p\,o\,i\,n\,t\,s-f\,i\,r\,s\,t\,4\,V\,e\,n\,d\,p\,o\,i\,n\,t\,s\right)$$

(2) Δ⁢y^P=l⁢a⁢s⁢t⁢ 4⁢P⁢e⁢n⁢d⁢p⁢o⁢i⁢n⁢t⁢s-f⁢i⁢r⁢s⁢t⁢ 4⁢P⁢e⁢n⁢d⁢p⁢o⁢i⁢n⁢t⁢s

We computed these values for the Veridical session as well, but Δŷ_*V*_ did not include subtraction from 70, since there was no 70 mm forward offset of the V target in the Veridical session.

To test whether perceiving a forward offset was linked to reduced recalibration in the Mismatch session, we compared the magnitudes of visual and proprioceptive recalibration between participants who reported perceiving a forward offset (*N* = 10) and those who did not (*N* = 52), in the Mismatch session. Recalibration was not normally distributed in these samples, so we used a non-parametric method (Wilcoxon rank-sum test, α of 0.05).

### Experiment 2

Apparatus, targets, and single trial procedure were identical to Experiment 1.

#### Trial blocks

In total, the experiment included 8 blocks, with each block containing 21 trials. V, P, and VP targets were presented in a repeating order throughout the experiment: VP, V, VP, P. In total, the experiment thus included 42 V trials, 42 P trials, and 84 VP trials. Visuo-proprioceptive offset was imposed gradually by shifting the white square 1.67 mm forward after each VP trial (every two trials), to a maximum offset of 140 mm at the end of Block 8.

#### Instructions

To test the participant’s awareness of the offset throughout the experiment, at the end of each block participants were first asked *“Did it always feel like the white square was directly on top of your left finger, or did it feel off?”* to screen out subjects who never noticed any offset. If participants replied with “it felt off,” they were asked in what direction the white square felt displaced from the left finger, and by how much at most. Both inches and centimeters were acceptable units. Only perceived offset magnitudes in the forward direction (true offset direction) were analyzed. All reported magnitudes were converted to centimeters. To prepare participants for this question being asked repeatedly, participants viewed task instructions in the form of a slideshow before beginning the experiment. This included the slides depicted in Figure 1C, which illustrate possible visuo-proprioceptive offsets people might perceive, without giving away that there would be a real offset and it would be in the forward direction (away from the participant).

#### Data analysis

Data consisted of the x,y coordinates of indicator finger endpoints and participants’ responses to the perceived offset question at the end of each trial block. Because the visuo-proprioceptive offset was imposed in the y (sagittal) direction, we computed participants’ mean estimate of V and P target position in the y-dimension for each block (*ŷ_*V(1)* …_*. *ŷ_*V(8)*_*, *ŷ_*P(1)* …_. y_*P(8)*_*). These estimates were computed by taking the average of the y-coordinate of indicator finger endpoints on V trials and P trials, respectively (10 or 11 trials, depending on block). Visual and proprioceptive recalibration (*△ŷ_*V*_, △ŷ_*P*_*) were calculated as:


(3) $$△\,{\hat{y}}_{V}=140-\left({\hat{y}}_{V\,\left(8\right)}-{\hat{y}}_{V\,\left(1\right)}\right)$$

(4) $$△\,{\hat{y}}_{P}={\hat{y}}_{P\,\left(8\right)}-{\hat{y}}_{P\,\left(1\right)}$$

Recalibration in the expected direction (i.e., overshoot for P targets and undershoot for V targets) comes out positive. Thus, a total recalibration (*△ŷ_*V*_* + *△ŷ_*P*_*) of 140 mm would indicate that 100% of the 140 mm offset was compensated for.

We analyzed perceived offset in the forward direction by converting all estimates to centimeters and computing the proportion of true offset that was perceived (i.e., at the end of block 4 there was a true offset of 7 cm, so a 3.5 cm perceived offset would be 50%).

To test whether perceived forward offset was related to total realignment, we computed Pearson’s correlation between maximum perceived forward offset and total realignment at both Block 4 and Block 8. When this was significant, we also computed Pearson’s partial correlations between maximum perceived forward offset and each of visual and proprioceptive recalibration, with α of 0.05.

### Experiment 3

#### Procedure

Participants were shown a series of thick white lines on a black background with the same apparatus as in the two prior experiments (Figure 1A). The lines varied in length (3.2, 6.2, 10.2, and 16.2 cm) and orientation (horizontal/lateral and vertical/sagittal) resulting in 8 combinations. In each trial, a random line combination was shown for 3 s. Visual noise was shown in between stimuli to reduce afterimage and make it difficult for participants to compare across trials. Each line combination was shown 6 times throughout the experiment, amassing a total of 48 trials. The appearance of a new line was prompted with an audio cue, and participants verbally reported their length estimate using either inches or centimeters. No performance feedback was given.

#### Data analysis

For each participant, we computed the mean estimated length of each of the eight line-orientation combinations and then converted it to a proportion by dividing estimated length by true length. At the group level, the Shapiro–Wilk test showed that the 3 and 6 cm estimated lengths were not normally distributed. To compare the proportion of each length perceived to 100%, the one sample Wilcoxon signed-rank test was used for the non-normally distributed lengths (3 and 6 cm), and one-sample *t* tests were used for the rest. All hypothesis tests were performed two-sided, with α of 0.05.

## Results

### Experiment 1

**FIGURE 2:**
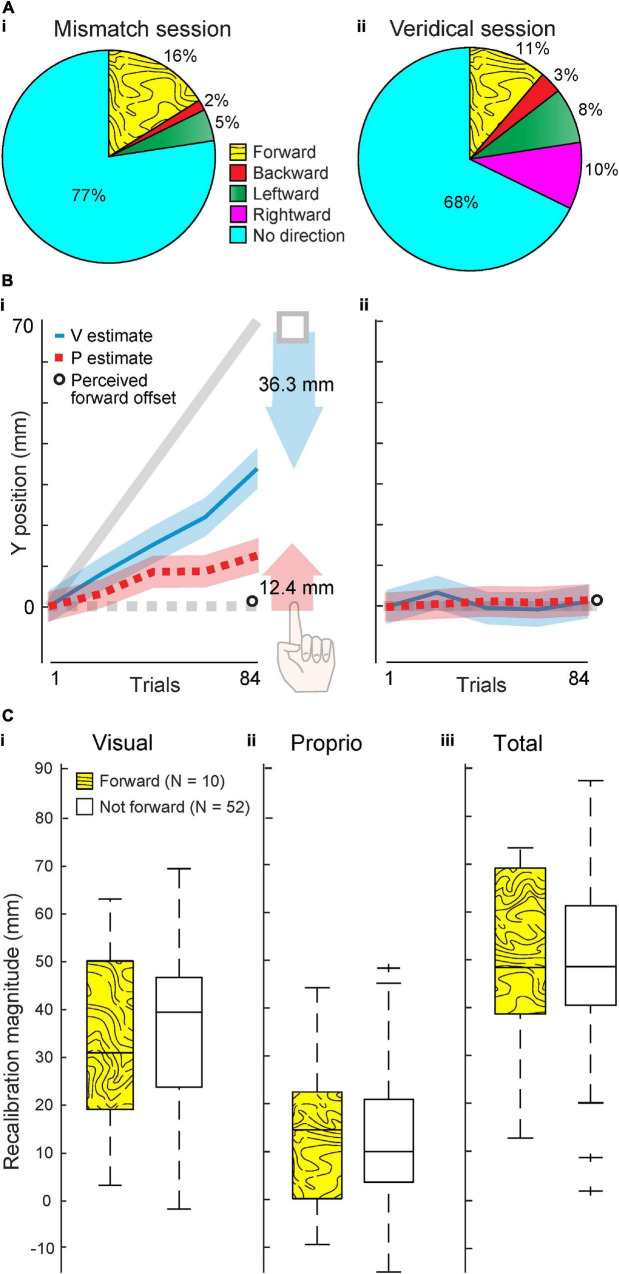
Experiment 1 results. **(A)** Percentage of 62 participants who reported perceiving an offset in various directions after the Mismatch (i) and Veridical session (ii). **(B)** Group visual and proprioceptive estimates (mean and standard error) across trials in the Mismatch **(i)** and Veridical (ii) session (*N* = 62). Shaded arrows reflect visual (blue) and proprioceptive (red) recalibration magnitude in the Mismatch session. Perceived forward offset magnitude, averaged across all 62 subjects, was less than 1 mm in both sessions (open circle). (C,i–iii) Visual, proprioceptive, and total recalibration in the Mismatch session, compared across subjects who did (*N* = 10) and did not (*N* = 52) report a forward offset at the end of the session. The central mark in each box indicates the median. Bottom and top edges of box represent 25th and 75th percentile, respectively. Dashed lines extend to most extreme data points not considered outliers, and crosses represent outliers.

In the Mismatch session, when a forward offset was imposed, 16% of 62 subjects (*n* = 10) reported a forward offset (Figure 2Ai). However, in the Veridical session, when no offset was imposed, 11% of the same 62 subjects (*n* = 7) reported a forward offset (Figure 2Aii). This between-session difference in proportion of individuals perceiving a forward offset was not statistically significant (χ^2^_1_ = 0.61, *p* = 0.43), which is inconsistent with the 70 mm forward offset in the Mismatch session being noticeable to subjects.

In the Mismatch session, subjects recalibrated vision 36.3 mm and proprioception 12.4 mm on average. This is 48.7 mm total, or 70% of the 70 mm offset (Figure 2Bi). Averaged across all subjects, perceived forward offset magnitude was less than 1 mm in each session (Figure 2B). Within the Mismatch session of Expt. 1, we also compared recalibration magnitude between subjects who reported a forward offset (*N* = 10) and those who did not (*N* = 52). These groups of participants did not differ significantly in visual recalibration (*W* = 1651, *p* = 0.81, Figure 2Ci), proprioceptive recalibration (*W* = 1619, *p* = 0.72, Figure 2Cii), or total recalibration (*W* = 1638, *p* = 1.0, Figure 2Ciii). These results do not support the idea that participants who perceived a forward offset recalibrated differently than those who did not perceive a forward offset.

### Experiment 2

**FIGURE 3:**
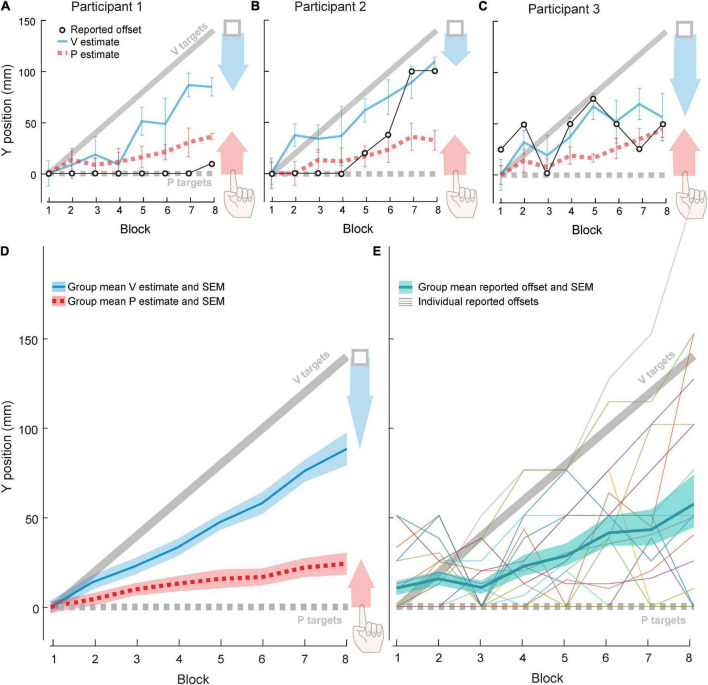
Experiment 2 results. Each block contained 21 trials: 10 VP and 5–6 each of V and P targets. **(A–C)** Three example participants. Red arrow represents proprioceptive recalibration (change in P target overshoot). Blue arrow represents visual recalibration (change in V target undershoot). Open circles represent forward offset reported after each block. **(D)** Group (*N* = 20) visual and proprioceptive estimates (mean and standard error) across blocks, relative to actual V targets (solid gray line) and actual P targets (dashed gray line). Shaded arrows reflect visual (blue) and proprioceptive (red) recalibration magnitude. **(E)** Group (*N* = 20) reported forward offset (mean and standard error) across blocks, relative to actual offset (solid gray line). Thin lines depict individual participants.

All participants used some combination of visual and proprioceptive recalibration to compensate for some portion of the 140 mm offset of the VP target. Three example participants (Figures 3A–C) were chosen to illustrate the range of recalibration observed across the group. On average, visual and proprioceptive recalibration increased with increasing offset, continuing to occur even after 70 mm of offset (Block 4) (Figure 3D).

We observed a wide range of patterns in participants’ perceived offset. Some detected no forward offset in most, if not all, of the experimental blocks. For example, Participant 1 (Figure 3A) did not report a perceived forward offset until the final block, and even then, they judged the forward offset to be a tenth of the actual value. Other participants reported an increasing offset magnitude across experiment blocks. For example, Participant 2 (Figure 3B) did not report a forward offset in the first four blocks but perceived an increasing forward offset across the final four blocks. Finally, some participants did not show any clear pattern. For example, Participant 3 (Figure 3C) increased and decreased their estimate of forward offset several times across blocks. At the group level, perceived offset was about 42% of actual offset across all eight blocks (Figure 3E).

**FIGURE 4:**
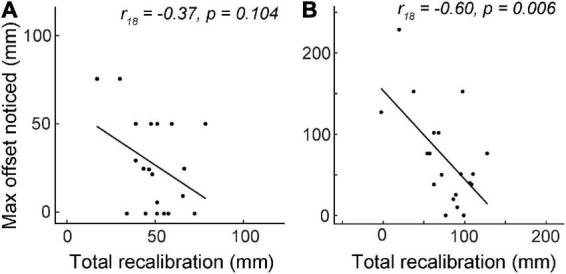
Experiment 2 total recalibration magnitude (visual plus proprioceptive) vs. the maximum visuo-proprioceptive offset reported. *N* = 20. **(A)** Between Block 1 and Block 4, there was no correlation between total recalibration and max noticed offset. At the end of Block 4, actual offset was 70 mm. **(B)** At the end of Block 8, total recalibration was negatively correlated with the maximum offset perceived.

In the first half of the experiment (Blocks 1–4), during which actual visuo-proprioceptive offset reached 70 mm, total recalibration was not significantly correlated with the maximum reported offset (*r*_18_ = −0.37, *p* = 0.10; Figure 4A). However, by Block 8, total recalibration was negatively correlated with max perceived offset (*r*_18_ = −0.60, *p* = 0.006), considered a large effect size (Cohen, 1988), suggesting that the more offset people noticed, the less they realigned overall by the time the mismatch reached 140 mm (Figure 4B). To determine whether this association might be driven more by differences in visual vs. proprioceptive recalibration, we also computed partial correlations between each of these variables and max perceived offset. After controlling for proprioceptive recalibration, visual recalibration was still negatively correlated with max perceived offset (partial *r*_17_ = −0.60, *p* = 0.006), and vice versa (partial *r*_17_ = −0.48, *p* = 0.039). This suggests that participants who perceived the greatest max offset had reduced recalibration in both visual and proprioceptive modalities.

### Experiment 3

**FIGURE 5:**
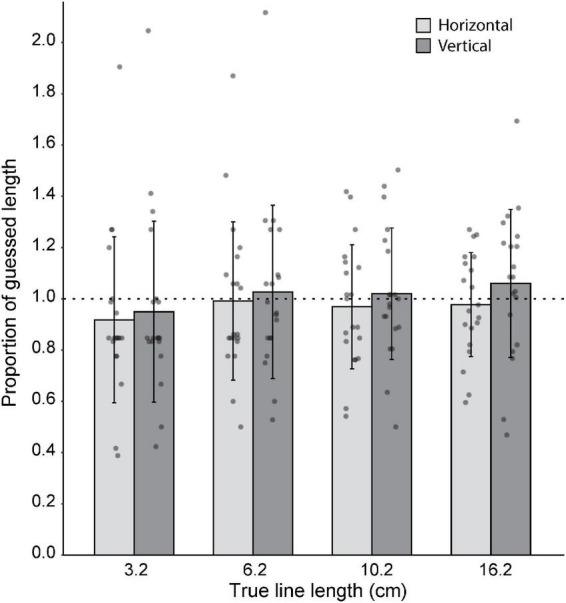
Group (*N* = 20) level estimates of line lengths. Gray dots represent the average length estimate per orientation for each subject. 1:1 proportion of guessed length is represented by the horizontal dotted line. For all except for the horizontal 3 cm line, there was no significant difference between guessed length and actual length. Error bars represent standard deviation.

In Experiment 3, participants’ ability to judge line lengths was examined. Overall, for both horizontal and vertical lines, participants were able to judge the lengths fairly accurately (Figure 5). One-sample Wilcoxon tests showed that line length estimates did not differ from true length for the vertical 3 cm line (*z* = −1.91, *p* = 0.056, median = 0.79), vertical 6 cm line (*z* = −0.64, *p* = 0.53, median = 0.93), or horizontal 6 cm line (*z* = −1.20, *p* = 0.23, median = 0.83), with *N* = 20 in each case. Similarly, one-sample t-tests showed that line estimates did not differ from true length for the vertical 10 cm line (*t*_19_ = −0.005, *p* = 0.99), horizontal 10 cm line (*t*_19_ = −0.95, *p* = 0.36), vertical 16 cm line (*t*_19_ = 0.74, *p* = 0.47), or horizontal 16 cm line (*t*_19_ = −0.77, *p* = 0.45). For all except for the horizontal 3 cm line, there was no difference between perceived and actual length. For the horizontal 3 cm line, participants underestimated the line length (*z* = −2.63, *p* = 0.009, median = 0.79, *N* = 20).

## Discussion

Here we asked how frequently participants perceive a forward visuo-proprioceptive mismatch, both spontaneously and after being asked to attend to visuo-proprioceptive alignment, and whether such awareness is linked to reduced recalibration. The results suggest three main conclusions. First, at small offsets (<70 mm), awareness of the offset does not often occur spontaneously (Figure 2A), but does occur after attention is directed to the possibility of an offset (Figure 3E). Second, when the offset is small, regardless of the perception, visuo-proprioceptive recalibration appears unaffected by awareness of the offset (Figures 2Bi, 3D). Third, when the offset is large (70–140 mm), greater awareness of the offset is associated with reduced recalibration (Figure 4B). We discuss these findings in relation to a causal inference framework.

### Conscious awareness of visuo-proprioceptive offset may require directed attention

We did not find evidence of participants spontaneously becoming aware of a gradual 70 mm visuo-proprioceptive offset in Experiment 1. These participants each completed two sessions on different days, in random order: One session with veridical visuo-proprioceptive calibration, and one with a gradual 70 mm forward offset. When questioned at the end of each session, 5% of participants reported perceiving a forward offset of any magnitude in the Mismatch compared to the Veridical session. However, the proportions of individuals who reported a forward offset in the two sessions did not differ statistically, suggesting that spontaneous awareness of this visuo-proprioceptive offset was uncommon. This result is not necessarily surprising. The gradual 70 mm offset was originally designed to be subtle enough that most individuals would not notice, while inducing a visuo-proprioceptive mismatch large enough that the brain would respond by recalibrating visual and proprioceptive estimates of hand position to compensate (Block and Bastian, 2011).

Experiment 2 was intended to make visuo-proprioceptive offset easier to perceive. Participants were instructed in advance that they would be asked about their perceived visuo-proprioceptive calibration, and they were asked to report their perceived offset at frequent intervals instead of only at the end. With these changes, most participants correctly reported a forward offset at some point in the session. In contrast with Experiment 1, 70% (14/20) of the subjects in Experiment 2 had reported a forward offset by Block 4. Thus, conscious awareness of a 70 mm visuo-proprioceptive offset may require directing participants’ attention to the calibration of visual and proprioceptive stimuli. This finding is in line with a causal inference framework. Based on this framework, knowledge of a common cause acts as a Bayesian prior and instructions directing attention toward a common cause may influence its perception (Chen and Spence, 2017). As such, asking the participants about their awareness leads to a larger probability that they would perceive a separate cause between the visual and proprioceptive cues.

### Visuo-proprioceptive recalibration reduced by awareness of offset at large offset magnitudes

An interesting finding is that when the offset was <70 mm, even though directing attention led to increased perception of an offset in Experiment 2, visuo-proprioceptive recalibration appears unaffected by this perception. Even with reported awareness of an offset, in the first half of Experiment 2 (0–70 mm offset), we did not detect a significant association between their max perceived offset magnitude and their total recalibration. Of course, we cannot rule out that there is a relationship (of moderate effect size: Cohen, 1988) that is too weak or noisy to detect in the present study, and that such a relationship might be detectable in a larger study. However, the Experiment 1 Mismatch session is consistent with a lack of relationship between perceived offset and recalibration. This also featured a 0–70 mm offset, and there was no indication that the individuals who reported any amount of offset in the correct direction recalibrated differently than the other participants.

Further support for the idea that perceived offset does not affect recalibration when the offset is <70 mm comes from comparing the magnitude of recalibration in the Mismatch session of Experiment 1 with recalibration at Block 4 in Experiment 2. At the end of the 70 mm Mismatch session of Experiment 1, subjects had recalibrated vision 36.3 mm and proprioception 12.4 mm on average. This is 48.7 mm total, or 70% of the 70 mm offset. In Block 4 of Experiment 2, subjects had recalibrated vision 37.0 mm and proprioception 13.1 mm. This is 50.1 mm in total, or 72% of the 70 mm offset. Thus, recalibration in the two experiments is almost identical, in total and in each modality, despite the greater awareness of the offset among Experiment 2 participants. Taken together, these results suggest that if offset is less than 70 mm, recalibration of vision and proprioception proceeds robustly even after the offset is recognized.

In contrast with smaller offsets (<70 mm), we found that when the offset is larger, awareness of the offset is clearly associated with reduced recalibration, consistent with the causal inference framework. In Experiment 2, max perceived offset was negatively correlated with total realignment by Block 8, when offset had reached 140 mm. The effect size of this correlation is considered large (Cohen, 1988). However, no association was evident at Block 4, when offset had reached 70 mm. It should also be noted that in Experiment 2, Block 8, visual realignment was 51.6 mm while proprioceptive realignment was 24.2 mm. This is 75.8 mm in total, or 54% of the 140 mm offset. Compared to the ∼70% compensation we observed in the first four blocks, this supports the idea that at larger magnitudes of mismatch, inferring a separate cause leads to reduced integration and recalibration, consistent with the causal inference framework.

### Linking these results with the causal inference framework

The causal inference literature makes clear predictions about cross-sensory recalibration in the context of offset awareness. In the case of visuo-proprioceptive recalibration, these predictions have been previously tested in experimental paradigms related to the rubber hand illusion (RHI) (Samad et al., 2015; Fang et al., 2019). The RHI involves a spatial discrepancy between the seen fake arm and the felt real arm that creates the illusion of body ownership over the fake arm when both arms are stroked synchronously (Botvinick and Cohen, 1998). This paradigm is thought to involve proprioceptive recalibration, usually described as drift (Butler et al., 2017). There are important differences between the RHI and the present study: our paradigm lacked any synchronous tactile stimulation, reduced the visual stimulus to a disembodied white square, and assessed visual as well as proprioceptive recalibration. However, the RHI can occur in the absence of synchronous stroking (Samad et al., 2015), so it is reasonable to compare our results with the RHI literature.

Samad et al. (2015) described the RHI as a consequence of causal inference involving three sensory stimuli: visual, tactile, and proprioceptive. When temporal visual and tactile signals are synchronous, and the distance between rubber and real hand is relatively small, a common cause is likely to be inferred (Samad et al., 2015). When a common cause is inferred, proprioceptive recalibration occurs in a predictable manner, and when separate causes are inferred, proprioceptive recalibration is reduced or eliminated (Samad et al., 2015; Fang et al., 2019). Our findings are thus somewhat contrary to the predictions of a causal inference framework. Recalibration was reduced at large visuo-proprioceptive offsets in Experiment 2 (up to 140 mm), and this reduction was indeed linked to perceived offset; however, at smaller offsets (<70 mm), sensory recalibration was similar between participants who perceived a common cause and those who did not. This was evident in both Experiment 1 and in the first half of Experiment 2.

Similar recalibration regardless of offset awareness suggests that explicit declaration of a separate cause may not override the intrinsic belief in a common cause at offsets of this magnitude. Indeed, others have suggested that unconscious belief in a common cause may continue even when subjects explicitly know about the offset (Welch and Warren, 1980; Chen and Spence, 2017). Specifically, knowledge of a relatively small prism-induced offset (10–16°) does not appear to affect proprioceptive recalibration (Welch and Warren, 1980). Thus, in our study, at offsets below 70 mm, participants could report perceiving a forward offset but still have an unconscious belief that both stimuli have a common cause.

One possible explanation for the apparent boundary at 70 mm of offset is participants’ own biased visual and proprioceptive estimates even in veridical conditions; even in the absence of perturbation, visual and proprioceptive finger estimates do not agree perfectly (Smeets et al., 2006). On average, these estimates are about 20 mm apart in healthy young adults (Liu et al., 2018). Interestingly, the average reported offset magnitude in people who perceived a forward offset in Experiment 2 was consistently less than half of the true magnitude. Thus, perceived offset was about 30 mm after the first half of Experiment 2, when true offset was 70 mm. This perceived offset is a roughly similar magnitude to the natural mismatch in visual and proprioceptive estimates (Liu et al., 2018). In other words, perhaps perceived offset must reach magnitudes substantially larger than a person’s own natural mismatch between visual and proprioceptive estimates in order to override their unconscious belief in a common cause. This could be tested in future studies by assessing whether an individual’s visuo-proprioceptive biases in veridical conditions (Liu et al., 2018) predict the offset magnitude at which awareness of the offset begins to reduce recalibration.

In addition, while visuo-proprioceptive recalibration differs in many respects from visuomotor adaptation—a process requiring feedback about movement errors—the concept of error attribution may be a relevant parallel (Berniker and Kording, 2008). It is possible that in the present study, when visuo-proprioceptive mismatch reaches the larger magnitudes (70–140 mm), the brain begins to attribute the mismatch to external sources (e.g., features of the VR apparatus or a shift in tactile marker position) as opposed to a mismatch between sensory estimates, resulting in less recalibration. The question of internal vs. external attribution is beyond the scope of the present study, which did not ask subjects who perceived an offset to explain what they attributed the offset to. Further studies would be needed to determine if visuo-proprioceptive recalibration is affected by attribution, as motor adaptation is.

### Neural overlap between multisensory spatial perception and attention systems

Attention is known to interact extensively with both sensory processing and behavioral performance. This includes regions known to be involved in multisensory integration, peripersonal space perception, and body ownership systems. Multisensory integration of visual and proprioceptive signals is largely associated with posterior parietal cortex (PPC). In monkeys, multimodal neurons responding to both “seen” and “felt” position of the limb exist in regions of PPC (Graziano, 1999; Graziano et al., 2000). Neuroimaging studies of the RHI have linked proprioceptive recalibration to PPC activity (Brozzoli et al., 2012). Recent human fMRI data indicates that visuo-proprioceptive congruence, a computation likely important for visuo-proprioceptive recalibration, modulates activity in several posterior parietal regions, such as anterior superior parietal lobule (Limanowski and Blankenburg, 2016), which corresponds to monkey area 5.

Human neuroimaging work has revealed distinct frontoparietal networks for peripersonal space perception, which is often associated with sensorimotor tasks, and for the subjective sensation of body ownership, which is linked to attention and awareness tasks (Grivaz et al., 2017). Functionally, the two networks mediate individual-environment interactions through their interactions within a more extended multisensory-motor frontoparietal network (Grivaz et al., 2017). For example, human neuroimaging studies have linked the feeling of hand ownership in the RHI with activity in premotor cortex (Ehrsson et al., 2004, 2005; Gentile et al., 2013). Recent work by Fang et al. (2019) has specifically linked neural activity in premotor cortex to RHI strength in monkeys. The study developed a linear probabilistic model that successfully predicted whether the fake arm would be integrated or segregated (suggesting inference of common cause vs. separate cause) at the level of single neurons in premotor cortex (Fang et al., 2019).

Attention allows the completion of behavioral goals through the flexible selection and enhancement of a set of sensory inputs, thereby increasing the strength of the neuronal signals within that sensory area (Clark et al., 2015). This is indicated by an increase in synaptic efficacy, decreases in neuronal response latency, and alterations to the neuronal receptive fields which may allow for more resources to be dedicated to the area of concern (Clark et al., 2015). Attention also increases motor performance outcomes. Dual-task studies suggest that divided attention results in the impairment of motor performance as attentional resources are being depleted (Song, 2019). We can assume that the repeated questioning about the participant’s perception after every block increased their attention to the possibility of an offset, which consequently allowed for more resources to be dedicated to the task, increasing their performance and perception of the offset.

### Limitations and future directions

When we found that participants consistently underestimated the magnitude of visuo-proprioceptive offset in Experiment 2, we wondered if this could be explained by participants being biased at estimating distances in general. For example, perhaps a participant actually perceives a 10 cm offset, but when asked to report that distance, estimates it to be only 4 cm. However, in Experiment 3, we found that participants were unbiased on average when asked to report the length of a series of white lines presented in the task display. This suggests that the under-reporting of perceived offset magnitude was not due to a systematic bias in estimating distances in general.

The conclusions of the present study are based on subjects’ verbal assessment of perceived offset, along with visuo-proprioceptive recalibration assessed by pointing with the other hand. One downside of relying on participants’ self-report of perceived offset is that participants may not be able to assess their perceptions accurately. Another could be a difference in the interpretation of questions, specifically the question “*did it always feel like the white square was directly on top of your left finger, or did it feel off?”.* This question was asked of every participant the same way, but some needed clarification before they could answer it. When necessary, we clarified that we were not asking about V or P trials or their right hand, but rather about their perception of their left hand during VP trials. Importantly, this was simply the first question, intended to screen out subjects who never noticed any offset. For those who responded that they did feel an offset, we then asked them to estimate the direction and magnitude, which is what was analyzed. It may be advantageous for future studies to assess these parameters by alternative methods such as gaze tracking, given the importance of eye movements in attention.

It should be noted that, although participants were asked to gaze at a red cross during each trial, the lack of eye tracking assessment in the present study means that we have no way to know where they were looking. Although participants heard a recording of this instruction at the beginning of every trial, we acknowledge it would not likely be sufficient to override a participant’s natural instinct to look toward the pointing target, at least initially. We must also offer the strong caveat that subjects may have employed different gaze strategies (e.g., gazing at the perceived P target location), which could explain substantial variations in offset detection or recalibration across participants.

We chose this method of assessing perceived offset in order for the results to be comparable with our previous investigations using this task (Munoz-Rubke et al., 2017; Mirdamadi et al., 2021). There are undoubtedly other methods that may yield interesting results in future studies. For example, if participants are asked to choose from an array of visual markers the one that best represents where the visual target was presented during the task, it might show that participants actually perceive more of the offset than they are consciously aware of. Taking a psychometric approach could also yield more precise estimates of participants’ perceptions with less chance of them misunderstanding a question. However, such procedures are more time consuming and would be difficult to repeat 8 times during a single-session experiment, as we did in Experiment 2.

Another manipulation that might affect awareness of offset would be to make the visuo-proprioceptive mismatch occur abruptly. In the present study, visuo-proprioceptive offset increased gradually, 1.67 mm per VP trial. If the 70 or 140 mm full offset were reached in one or just a few trials, we might suppose that most participants would become aware of the perturbation. On the other hand, an abrupt shift may seem more natural and requiring of fewer cognitive resources; in real life, when we experience such an offset by viewing our hand under water, the visuo-proprioceptive offset occurs abruptly, not gradually.

In a Bayesian causal inference framework (Ghahramani et al., 1997; Samad et al., 2015), we would expect participants with more precise visual and proprioceptive estimates to more easily detect an offset between visual and proprioceptive cues. In theory, we could test this prediction in a future study with a large baseline block of veridical visual and proprioceptive targets, which would allow us to estimate participants’ visual and proprioceptive variance (Block and Bastian, 2010). In practice, this prediction could be complicated by the presence of participants’ naturally-occurring biases in visual and proprioceptive target estimation (Smeets et al., 2006; Liu et al., 2018), as discussed above. In other words, a participant may have low variance in their visual and proprioceptive estimates, but perceive these stimuli as several centimeters apart even when presented veridically. This person may be worse at detecting a true offset, because they are already accustomed to their own biased perception. Or it may depend on the spatial orientation of their natural biases. In any case, this would be an interesting question for future study.

### Conclusion

Here we found that when a 70 mm mismatch is gradually imposed between visual and proprioceptive cues of hand position, individuals are unlikely to become aware of this spontaneously. When directed to attend to visuo-proprioceptive alignment by repeated questioning, conscious awareness of the mismatch was linked to reduced compensation only at higher mismatch magnitudes (70–140 mm). These results are consistent with causal inference predictions at larger offsets. At smaller offsets, conscious perception of an offset may not override unconscious belief in a common cause, perhaps because the perceived offset magnitude is in range of subjects’ natural sensory biases.

## Data availability statement

The raw data supporting the conclusions of this article will be made available by the authors, without undue reservation.

## Ethics statement

The studies involving human participants were reviewed and approved by Indiana University Human Research Protection Program Board Affiliation: Indiana University. The patients/participants provided their written informed consent to participate in this study.

## Author contributions

AH: conceptualization, investigation, formal analysis, and writing—original draft. TL-M: formal analysis, software, visualization, and writing—review and editing. HB: conceptualization, methodology, software, writing—review and editing, funding acquisition, and supervision. All authors contributed to the article and approved the submitted version.
